# Exploration of Genetic and Genomic Resources for Abiotic and Biotic Stress Tolerance in Pearl Millet

**DOI:** 10.3389/fpls.2016.02069

**Published:** 2017-01-23

**Authors:** Radha Shivhare, Charu Lata

**Affiliations:** ^1^National Botanical Research Institute (CSIR)Lucknow, India; ^2^Academy of Scientific and Innovative ResearchNew Delhi, India

**Keywords:** diversity, downy mildew, germplasm, marker-assisted breeding, panicoid, quantitative trait loci, stress tolerance, terminal drought

## Abstract

Pearl millet is one of the most important small-grained C_4_ Panicoid crops with a large genome size (∼2352 Mb), short life cycle and outbreeding nature. It is highly resilient to areas with scanty rain and high temperature. Pearl millet is a nutritionally superior staple crop for people inhabiting hot, drought-prone arid and semi-arid regions of South Asia and Africa where it is widely grown and used for food, hay, silage, bird feed, building material, and fuel. Having excellent nutrient composition and exceptional buffering capacity against variable climatic conditions and pathogen attack makes pearl millet a wonderful model crop for stress tolerance studies. Pearl millet germplasm show a large range of genotypic and phenotypic variations including tolerance to abiotic and biotic stresses. Conventional breeding for enhancing abiotic and biotic stress resistance in pearl millet have met with considerable success, however, in last few years various novel approaches including functional genomics and molecular breeding have been attempted in this crop for augmenting yield under adverse environmental conditions, and there is still a lot of scope for further improvement using genomic tools. Discovery and use of various DNA-based markers such as EST-SSRs, DArT, CISP, and SSCP-SNP in pearl millet not only help in determining population structure and genetic diversity but also prove to be important for developing strategies for crop improvement at a faster rate and greater precision. Molecular marker-based genetic linkage maps and identification of genomic regions determining yield under abiotic stresses particularly terminal drought have paved way for marker-assisted selection and breeding of pearl millet cultivars. Reference collections and marker-assisted backcrossing have also been used to improve biotic stress resistance in pearl millet specifically to downy mildew. Whole genome sequencing of pearl millet genome will give new insights for processing of functional genes and assist in crop improvement programs through molecular breeding approaches. This review thus summarizes the exploration of pearl millet genetic and genomic resources for improving abiotic and biotic stress resistance and development of cultivars superior in stress tolerance.

## Introduction

Pearl millet [*Pennisetum glaucum* (L.) R. Br.] (2n = 2x = 14) is an important tropical C_4_ small-grained cereal crop that belongs to family Poaceae and sub family Panicoideae. It is a highly tillering, cross-pollinating millet crop with a short life cycle and a large genome size (∼2352 Mbp) (Bennett et al., 2000). It is usually grown in marginal environments of arid and semi-arid regions of Sub-Saharan Africa and the Indian subcontinent characterized by scanty and erratic rainfall, poor soil conditions and high temperature where staple cereals such as rice, wheat, maize, and even sorghum are likely to fail (Vadez et al., 2012). It is the sixth most economically important and promising cereal crop for providing food security to almost 90 million poor people inhabiting across the high temperate regions of Africa and Asia^1^. It is a dry land crop and can withstand poor environmental conditions such as low moisture, high temperature, and nutrient deficient soil where other cereal crops could not hold up their growth. It is cultivated principally for grain production but it also cultivated by marginal farmers for hay, bird feed, fuel, and forage materials. Globally, it is cultivated on approximately 31 million ha of land and contributes around 50% of total global millet production^2^. In India, it is cultivated in an area of approximately 7.8 million ha with an average of about 9.25 million tons of grain production^3^. However, in the year 2013–2014, India was the largest producer of pearl millet (10 mt) followed by Nigeria (5 mt) (Malik, 2015). Pearl millet grains are high in nutrient composition and are considered an inexpensive source of energy as compared to staple cereals such as wheat, rice and maize in terms of micronutrients (Zinc and Iron) (Kumar et al., 2016), protein content and amino acid composition for the resource-poor farmers (Kanatti et al., 2014). In fact, pearl millet is superior in protein content, quality, protein energy ratio, and metabolizable energy levels as compared to sorghum (Vadez et al., 2012). Thus millions of people depend on this crop for their dietary needs and livelihood^2^. Furthermore, pearl millet has also been long known as a potential biofuel crop (Wu et al., 2005; Lata, 2015).

Pearl millet accessions exhibit a wide range of phenotypic and genotypic variations for various agronomic and stress tolerance traits owing to its cultivation in varied agro-climatic conditions and soil types across the world. Despite having a wide and diverse germplasm collection and genetic resources, pearl millet was until recently considered an orphan crop and only a narrow range of its germplasm could be exploited for improving its valuable agronomic traits, stress tolerance, and productivity (Passot et al., 2016). In the past few years most of the allele mining studies in this crop for improvement of various agronomic traits such as yield and stover quality as well as for abiotic and biotic stress resistance such as drought tolerance (DT) and downy mildew resistance have been achieved by generating and using bi-parental mapping populations (Vengadessan et al., 2013). Genetic maps based on molecular markers and identified quantitative trait loci (QTLs) have been used for developmental and breeding programs in pearl millet to enhance its productivity under the present scenario of global climatic change. Molecular markers like restriction fragment length polymorphism (RFLP), amplified fragment length polymorphism (AFLP), expressed sequence – simple sequence repeats (EST-SSRs), conserved intron spanning primer (CISP), single strand conformational polymorphism-single nucleotide polymorphism (SSCP-SNP) and diversity array technology (DArT) are dominant and proven to be useful in the evaluation of QTLs associated with various traits (Jones et al., 1995, 2002; Yadav R.S. et al., 2003; Bidinger et al., 2007), terminal drought stress tolerance and adaptation (Yadav R.S. et al., 2004; Kholova et al., 2012) and disease resistance (Hash et al., 2006a; Jogaiah et al., 2014). Along with genetic markers, core or mini core and reference collections are also helpful to identify new sources of abiotic and biotic stress resistance in pearl millet.

Though, conventional linkage mapping has helped in identifying different quantitative traits associated with stress tolerance in pearl millet but these are limited by the resolution provided by two parent-derived mapping populations (Dwivedi et al., 2012). Association mapping, also known as linkage disequilibrium (LD) mapping opens a new platform for allele mining with the help of ancestral recombination events in natural populations or germplasm collections to make marker-phenotype associations. It has the advantage over QTLs associated linkage mapping in terms of less laborious, time efficient and generation of 1000s of recombinants with large and diverse gene pool. It has been widely used in many crops including maize, barley, sorghum, rice, and common wheat to detect important markers or genes associated with abiotic and biotic stress resistance (Rajaram et al., 2010, 2013; Senthilvel et al., 2010; Sehgal et al., 2012, 2015; Jogaiah et al., 2014). Recently a pearl millet inbred germplasm association panel (PMiGAP) of about 346 lines was generated that represented a collection of approximately 1000 diverse cultivars, landraces and parents of mapping population from various regions of Africa and Asia, out of which 250 lines were used for association mapping of DT traits (Sehgal et al., 2015). This PMiGAP is thought to provide new insights for fine mapping of QTLs and allele mining of favorable genes for important agronomic traits.

Apart from genomics tools, various advanced technologies such as high throughput sequencing, insertional mutagenesis, targeted induced local lesion in genomes (TILLING), gene silencing, genome editing, and transgenics may also play important role in improving our understanding of complex biological mechanisms of stress regulation, adaptation, and resistance. Huge datasets obtained from various omics technologies could be useful for identifying candidate genes/proteins that can be incorporated in crop improvement programs for developing cultivars superior in stress tolerance. Until now there have been limited reports on identification and exploitation of potential genes for abiotic tolerance or biotic stress resistance in pearl millet (Girgi et al., 2006; Latha et al., 2006; Mishra et al., 2007; Agarwal et al., 2008). It is anticipated that the ongoing pearl millet genome sequencing project will be very useful for exploring its genetic and genomic resources for crop improvement programs directed toward improving important agronomic traits such as yield and stress resistance. This review thus primarily focuses on the sources of tolerance to abiotic and biotic stresses, agronomic traits of importance, as well as efforts to identify new sources of variation in pearl millet germplasm collections and to promote these resources for genomics/marker- assisted breeding program(s) for product development related to food, feed, and bioenergy traits in pearl millet.

## Morphology, Origin, and Domestication

Pearl millet is a robust erect highly cross-pollinated annual C_4_ grass with slender culms and profuse root system and the plant may reach up to a height of 4 m. However, improved open-pollinated varieties (OPVs) and hybrids are reportedly shorter^1^ (Manga and Kumar, 2011). Its leaves are minutely serrated alternate, simple, flat, green with linear blades that can be up to 1.5 m long and 8 cm wide while its inflorescence is a 12–30 cm long panicle with a circumference of 7–9 cm that bears the seeds^4^. Involucres composed of bristles each of which enclosing 1–9 spikelets is one of the most characteristic features of *Pennisetum glaucum* (de Wet et al., 1992). There could be around 500–3000 spikelets on a panicle depending upon the variety. Pearl millet seeds are small (0.5–3 mm long and each could weigh between 0.003 and 0.02 g), wedge-shaped to spherical and can be of various colors ranging from white or yellow, brown or even purple^5^. Seeds could attain physiological maturity within 27–30 days after 50% flowering^6^. It is well-adapted to sandy or light loam well-drained soils and has a good DT capacity. It could be cultivated from late May to September and high temperature is a prerequisite for its optimum growth^5^. Pearl millet could broadly be divided into two morphotypes namely, tall and dwarf. Dwarf types are usually leafier and are commonly used for grazing.

Pearl millet is thought to be originated in Dhar Tichitt, a Saharan site in Mauritania of West Africa about 3500 B.C. ago (Amblard and Pernès, 1989). The findings of an archeologist Birimi, confirms the Sahara and Sahel hypothesis of origin and their widespread distribution and cultivation of wild and cultivated pearl millet across sub-Saharan Africa (Amblard and Pernès, 1989; D’Andrea and Casey, 2002). de Wet et al. (1992) reported pearl millet to be a native along the southern fringes of Sahara with a distribution across semi-arid tropics of Africa, and also suggested its introduction as a cereal crop into the South Asian continent. It has been introduced in India since 2000 B.C., in the USA in the 1850s and in Brazil in the 1960s (Garí, 2002; Oumar et al., 2008; Rai et al., 2013). Across different regions of the world, pearl millet is called by various common names including *geohatsii. mahangu, sanio, babala*, etc. in different areas of Africa; *milheto kaustubh* in Brazil; *bajri, sajje, kambu, bajra* in different states of India; *miglio* in Italian, *grano* in Span, *Type de graine* in France, etc. (Malik, 2015). With approximately 140 species, *Pennisetum* is the largest genera in the Paniaceae tribe with diverse ploidy and reproductive behavior^7^ (**Table 1**). Brunken et al. (1977) categorized *Pennisetum* into two reproductively isolated species, namely *P. purpureum* Schumach (Napier grass) (2n = 4x = 28) and *P. americanum* (syn. *P. glaucum*) (2n = 2x = 14). Napier grass is perennial and is found across wet tropics of the world while *P. americanum* is annual and is a native of semi-arid tropics of Africa and India. *P. americanum* has been classified into three distinct morphological units or subspecies (i) wild plants Subsp., *violaceum (monodii)* – that neither show any characteristic of a cultivated plant nor are dependent upon man for their survival; (ii) weedy plants (Subsp., *stenostachyum*) – that exhibit intermediate morphology and are exclusively associated with pearl millet cultivation and are also indirectly dependent upon man; and (iii) cultivated plants (Subsp., *glaucum*) – that show distinctly stalked and involucres persistent at maturity, and are directly dependent upon agricultural activities taken up by man for their survival. Together these three subspecies displayed three distinct adaptive strategies in response to the selective pressures thrust upon them during domestication. *P. glaucum* is the only cultivated species used for seed purpose while *P. purpureum* and its hybrids with pearl millet are usually cultivated for fodder in different parts of the world^7^.

**Table 1:** Details of genome organization and other characteristics of significant cultivated and wild *Pennisetum* species.

Species	Chromosome no.	Ploidy	Reproductive behavior	Mating type	Life cycle	Reference
*P. alopecuroids*	2n = 2x = 18	Diploid	Sexual	Inbreeder	NA	Harlan and de Wet, 1971; Jauhar, 1981; Khalfallah et al., 1993; Martel et al., 1997; http://cropgenebank.sgrp.cgiar.org
*P. glaucum*	2n = 2x = 14	Diploid	Sexual	Inbreeder	Annual	
*P. mezianum*	2n = 4x = 32	Tetraploid	Apomictic	Inbreeder	Perennial	
*P. mollissimum*	2n = 2x = 14	Diploid	Sexual	Inbreeder	Annual	
*P. nohenacken*	2n = 2x = 18	Diploid	Sexual, apomictic	Inbreeder	Perennial	
*P. orientale*	2n = 4x = 36	Tetraploid	Apomictic	Inbreeder	Perennial	
*P. pedicellatum*	2n = 6x = 54	Hexaploid	Apomictic	Inbreeder	Perennial	
*P. polystachyon*	2n = 6x = 54	Hexaploid	Apomictic	Inbreeder	Annual	
*P. purpureum*	2n = 4x = 28	Tetraploid	Sexual	Inbreeder	Perennial	
*P. ramosum*	2n = 2x = 10	Diploid	Sexual, apomictic	Inbreeder	Annual, biennial	
*P. schweinfurthii*	2n = 2x = 14	Diploid	Sexual	Inbreeder	Annual	
*P. setaceum*	2n = 3x = 27	Triploid	Apomictic	Inbreeder	Perennial	
*P. setaceum*	2n = 6x = 54	Hexaploid	Apomictic	Inbreeder	Perennial	
*P. squamulatum*	2n = 6x = 54	Hexaploid	Apomictic	Inbreeder	Perennial	
*P. villosum*	2n = 4x = 36	Tetraplod	Apomictic	Inbreeder	Perennial	
*P. violaceum*	2n = 2x = 14	Diploid	Sexual	Inbreeder	Annual	


## Nutritional Value and Medicinal Uses

Pearl millet has an excellent nutritional composition and is a rich source of energy (361 Kcal/100 g) as compared to staple cereals like rice (345 Kcal/100 g) and wheat (346 Kcal/100 g) (Malik, 2015). Further with regard to nutritional quality, it is generally equivalent to maize and superior to sorghum in terms of protein content, quality, efficiency ratio, and metabolizable energy (Vadez et al., 2012). Although it is deficient in essential amino acids, it contains 35% more lysine as compared to sorghum (Rooney and McDonough, 1987). Additionally, it also does not contain condensed polyphenols which are a major cause of decreased digestibility, for example, tannins in sorghum. Pearl millet grains contain 5–6% oil and are also rich in important micronutrients like iron and zinc (Jambunathan and Subramanian, 1988; Malik, 2015). Hence, pearl millet significantly contributes toward protein, iron, and zinc uptake as well as serve as the cheapest source of energy to low-income group consumers of semi-arid tropics including India.

Furthermore, pearl millet has plenty of health-promoting attributes owing to its nutritional composition and hence has an immense potential toward medicinal uses. As for example, its high fiber content can make it a potential component in the diets of patients suffering from constipation, obesity, and gallstones (Malik, 2015). Also since pearl millet grains are gluten free and have low glycemic index, they can be very beneficial for persons with celiac disease and/or diabetes. Further due to its anti- or hypo-allergic properties, it can be safely incorporated in the diets of pregnant women, infants, lactating mothers, elderly and convalescents. Its flour can be conveniently used for making chapattis, porridge or can be used in the boiled or roasted form or even as weaning mixtures. Pearl millet grains are also locally brewed in Africa and Asia to produce alcoholic or non-alcoholic beverages (Dwivedi et al., 2012). Thus pearl millet grains have immense medicinal value and should be aggressively promoted by dieticians and nutritionists so that a large section of society could be benefited.

## Germplasm Collections and Diversity Assessment

*Ex situ* seed storage is one of the most extensively used methods for conserving pearl millet genetic resources (Dwivedi et al., 2012). Germplasm collections help in maintaining the genetic resources so that they can be used for crop development and improvement programs globally. Pearl millet germplasm have been preserved in various national and international gene banks. India with 11243 accessions has the largest collection of pearl millet germplasm followed by Brazil (7225) and Canada (3764) (Dwivedi et al., 2012). The initiative for the conservation of pearl millet genetic resources in India was taken up by International Crop Research Institute for Semi-Arid Tropics (ICRISAT), Patancheru in collaboration with different national and international organizations including several national organizations such as National Bureau of Plant Genetic Resources (NBPGR), All India Coordinated Pearl Millet Improvement Project (AICPMIP), National Agricultural Research Systems (NARS), Agricultural Universities, and other ICAR Institutes etc. About 65 organizations had contributed approximately 10,764 accessions of pearl millet in different disciplines of ICRISAT, in past few years. Major international contributors for pearl millet germplasm are Institute Francais de Recherche Scientifique pour le development en Cooperation (ORSTOM), France (2,178); Rockefeller Foundation, New Delhi, India (2,022); and International Bureau for Plant Genetic Resources (IBPGR), Rome, Italy (974) at ICRISAT. ICRISAT has a total of 21,594 pearl millet accessions from 51 countries, thus becoming the largest center for pearl millet germplasm collection in the world (Upadhyaya et al., 2007; Upadhyaya et al., 2015) (**Table 2**). ICRISAT also maintains some of the pearl millet wild relatives in an *ex situ* field gene bank at its Patancheru campus (Dwivedi et al., 2012). Conservation of wild relatives is also very important as they could contribute beneficial traits to the cultivated gene pool, for example, a few accessions of *P. glaucum* subsp. *monodii* namely PS# 202, 637, 639, and 727 are good sources of resistance to a cereal parasitic weed, *Striga hermonthica*, in sub-Saharan West Africa. PS 202 is also a source of resistance to one of the most devastating fungal diseases of pearl millet, downy mildew (Wilson et al., 2004). A few wild accessions could serve as a source for rust resistance (Hanna and Dujardin, 1985) or also as alternative systems for cytoplasmic male sterility (Hanna, 1989). Thus a more detailed exploration for useful traits from pearl millet wild relatives is required to be carried out.

**Table 2:** Geographical distribution of pearl millet germplasm accessions assembled at ICRISAT gene bank (as on 1 January 2007; Upadhyaya et al., 2015).

	Number of accessions		
		
Country	Cultivated	Wild	Total
**Africa**			
Burkina Faso	862	5	867
Cameroon	911	85	996
Central African Republic	142	10	152
Ghana	283	–	283
Malawi	298	12	310
Mali	1048	109	1157
Namibia	1118	10	1128
Niger	1130	178	1308
Nigeria	2064	10	2074
Senegal	393	12	405
South Africa	162	3	165
Sudan	587	27	614
Tanzania	478	25	503
Togo	520	–	520
Uganda	118	1	119
Zambia	155	7	162
Zimbabwe	1384	13	1397
Others	447	76	523
**Asia**			
India	7835	145	7980
Lebanon	108	–	108
Yemen	290	32	293
Others	28	1	30
**Europe**	45	1	46
**Americas**			
USA	219	10	229
Others	12	1	13
**Oceania**	8	–	8
**Total**	20844	750	21594


Descriptor list has also been developed and used to characterize pearl millet (IBPGR and ICRISAT, 1993) germplasm for various morphological and agronomic traits (Dwivedi et al., 2012). The information so generated along with passport data has been used to assess patterns of diversity in germplasm collection, as a result of which several interesting information regarding their utility in pearl millet genetics and breeding could be obtained. As for example, Stich et al. (2010) reported patterns of diversity in flowering, photoperiod response, panicle length and population structure differentiation in 145 inbreds derived from 122 Western and Central African landraces. These landraces also exhibited exceptional buffering capacity against variable environmental conditions while the landraces from Cameroon, Togo, and Ghana were found to be good sources of earliness and bigger seeds. On the other hand, landraces from Yemen were found to be potential sources of variation for early maturity, short stature, large seeds, and cold tolerance. Similarly, Yadav and Bidinger (2008) evaluated 169 landraces from India for their grain and stover yield and discerned significant differences among them for the examined parameters such as early flowering, profuse tillering, more panicles and larger seeds. In fact, several landraces also outperformed controls in terms of both grain and stover yield and were thus considered as potential resources for developing dual-purpose hybrids adapted for arid climatic conditions. Upadhyaya et al. (2007) evaluated 20,844 accessions from 51 countries for 23 traits including number of flowering days, plant height, tiller number, 100 seed-weight, panicle length and shape, seed shape and color, and reported significant variations among the accessions studied. Therefore, germplasm collection (both cultivated and wild) and their characterization using both conventional and advanced techniques such as TILLING need to be done for expansion of its cultivated gene pool.

## Developing Core, Mini-Core, and Reference Sets

Pearl millet exhibits a vast range of genetic diversity in its global germplasm collection. Core and mini-core collections of pearl millet germplasm could serve as wonderful cost effective resources for allele mining and identification of genotypic variants for resistance to biotic and abiotic stresses as well as other important agronomic traits. A core collection comprises of about 10% of the entire germplasm collection while a mini core is comprised of ∼1% of the total collection. Upadhyaya et al. (2009) reported a core collection of 2094 pearl millet accessions which was huge in itself to be evaluated by breeders for crop improvement. Hence, a mini-core collection of 238 pearl millet accessions representing all accessions of the core collection was constituted using data on 10 qualitative and eight quantitative traits (Upadhyaya et al., 2011). This mini-core collection with its greatly reduced size could be effectively utilized for accurate evaluation of desirable traits including tolerance to abiotic and biotic stresses as well as for mapping with molecular markers and identification of trait-specific germplasm and discovery of candidate genes (Upadhyaya et al., 2011; Manga, 2015). Other than the core and mini- core collections, a genotype based reference set comprised of 300 accessions is also available in pearl millet (Upadhyaya et al., 2011). A global composite collection of accessions in pearl millet based on SSRs and high-throughput assays has also been developed at ICRISAT prior to the formation of reference sets for assessing population structure and diversity (Dwivedi et al., 2012). This reference set reportedly capture 87–95% of the allelic diversity of the composite collections (ICRISAT, 2008/2009). However, a persistent research needs to be directed in order to maintain up-to-date pearl millet core and mini- core collections.

## Identification of Pearl Millet Germplasm for Stress Tolerance

### Tolerance to Abiotic Stresses

Abiotic stresses adversely affect all crops by negatively impacting their growth and yield potential, and pearl millet is also no exception to it. In fact, it is also vulnerable to various abiotic stresses like other crops despite the fact it is generally considered to be well-adapted to drought, salinity, high temperature, lodging, and poor soil conditions (Zegada-Lizarazu and Iijima, 2005). Therefore, it is very imperative that identification and utilization of genetic variations for abiotic stress tolerance in pearl millet could be helpful in enhancing its adaptation to various stresses. Considering a large collection of pearl millet germplasm (both cultivated and landraces) in various national and international gene banks, there is a huge opportunity for researchers to identify and characterize the hidden DNA stretches responsible for abiotic stress tolerance which would eventually be beneficial in speeding up the crop improvement programs. Further accurate phenotyping along with improved understanding of the physiological and molecular basis of abiotic stress tolerance in this crop will be helpful in identifying and utilizing new potential candidate genes for developing varieties with superior stress tolerance. Among various millets, pearl millet has received comparatively more attention toward the identification of sources of tolerance to drought, salinity, and high temperature (Dwivedi et al., 2012). ICRISAT even has developed phenotypic screens for various abiotic stresses for identifying tolerant germplasm (Krishnamurthy et al., 2007; ICRISAT, 2008/2009). Identification and exploitation of pearl millet germplasm for abiotic stress tolerance is discussed in following sections.

#### Drought

Pearl millet is a DT crop and substantial work has been done to understand its response and adaptation to water deficit conditions at different developmental growth stages. Further impact of low water stress conditions depends on growth stages of the crop. Water stress during germination stage or seedling emergence stages cause seedling death resulting in poor crop establishment (Farooq et al., 2009; Lata et al., 2015). Severe moisture stress at seedling stage is the major cause of low yield of pearl millet in the semi-arid regions (Carberry et al., 1985; Soman and Peacock, 1985; Soman et al., 1987). It has been found that after the seedling establishment, the impact of the drought stress has little effect on pearl millet grain yield (Lahiri and Kumar, 1966). Effect of drought stress on the germination of seedling depends on the availability of water and it has been shown that impact of low water stress is closely related to leaf formation and secondary root development (Gregory, 1983). The impact of moisture stress has also been analyzed using varying concentrations of polyethylene glycol (PEG) 6000 and found that it significantly affected various seedling parameters such as germination percentage, root and shoot length, and root/shoot ratio (Govindaraj et al., 2010). Use of PEG 6000 for screening pearl millet germplasm at germination and early seedling growth stages was also established as a simple, rapid and cost-effective method. The study revealed TNBH 0538, TNBH 0642, and ICMV-221 genotypes as better performers under all four imposed moisture stress regime while PT6034 was found to be least resistant to moisture stress. Drought stress at vegetative phase exhibit little or almost negligible reduction in crop growth and yield owing to its asynchronous tillering and rapid growth rate which allow it to recover quickly (Bidinger et al., 1987; Mahalakshmi et al., 1987). Further drought stress during the vegetative phase delays the flowering time of the main shoot. This phenological plasticity gives it a chance to escape from the most sensitive flowering phase until the stress has been relieved (Henson and Mahalakshmi, 1985). However, post-flowering drought stress or terminal drought stress impose the most drastic impact on pearl millet grain and stover yield as well as on yield stability (Mahalakshmi et al., 1987; Winkel et al., 1997; Bidinger and Hash, 2004; Kholová and Vadez, 2013). It has been reported that early flowering pearl millet cultivars having few but effective basal tillers, low biomass, and high harvest index (including panicle harvest index) can effectively overcome the effects of terminal drought stress over other genotypes (Yadav O.P. et al., 2003; Bidinger et al., 2005). Further in order to understand whether control of water loss under non-limiting water conditions is implicated in terminal DT of pearl millet, a study was carried out involving two contrasting pair of parents of mapping population (PRLT2/88-33 X H77/833-2 and 863B-P2 X ICMB 841-P3) and near-isogenic lines (NILs) generated from introgression of a major DT-QTL from PRLT2/88-33 (donor) into H77/833-2 (NILs-QTL) under water deficit conditions. Tolerant varieties and NILs-QTL showed low transpiration rate as compared to sensitive genotypes in well-watered conditions (Kholova et al., 2010a). Kholova et al. (2010b) established that pearl millet genotypes carrying a DT-QTL for terminal DT, including tolerant and NIL-QTLs, have higher ABA levels and lower transpiration rate (Tr) under well-watered conditions as compared to sensitive genotypes at all vapor pressure deficit (VPD) levels, ultimately contributing to absolute water saving during grain-filling. Furthermore, F_7_ recombinant inbred lines (RILs) from a cross between 863B X ICMB 841, were used to identify four QTLs that contributed to increased Tr under high VPD conditions, out of which the major QTL was mapped on LG 6 (Aparna et al., 2015). This study also provided an additional evidence for a link between DT and water savings from lower Tr under high VPD.

Across different regions of West Africa, Ghana, and North Western India, a number of landraces have been identified which could serve as a rich source of diversity for abiotic stress tolerance in pearl millet (**Table 3**). These landraces because of their high grain and stover yield as compared to the conventionally bred lines are preferentially cultivated in arid zones of northwestern India (Gujarat, Rajasthan, and Haryana) where drought is a common phenomenon (Bidinger et al., 2009). As for example, CZP9802, the first open pollen pearl millet variety derived from the landraces of Rajasthan is not only highly adapted to drought but is also high yielding (14–33% higher grain yield and 18–36% higher stover yield) as compared to the controls Pusa 266 and ICTP 8203 (Yadav, 2004; Dwivedi et al., 2012). CZP9802 is endowed with the property to escape terminal drought stress owing to its exceptional attributes of flowering within 48 days and maturity in 75 days making it a suitable cultivar for arid zones of India (Yadav O.P. et al., 2004). Further Okashana 1, another early maturing variety is very popular in Namibia (Daisuke, 2005). Similarly, a relatively photoperiod insensitive early maturing West African landrace *Iniadi* with compact, conical panicles and bold grains has significantly contributed toward the development of several pearl millet cultivars worldwide including ICMV 88904 (released as ICMV 221) (Andrews and Kumar, 1996; Witcombe et al., 1997). ICMV 221 which is being cultivated in India and several African countries is terminal DT, downy mildew resistant and has improved grain yield potential.

**Table 3:** Available genotypes for abiotic and biotic stress tolerance in pearl millet.

Abiotic stress		Reference
Drought	CZP 9802; 863B, PRLT 2/89-33ICMP 83720, ICMV 9413, and ICMV 94472	Yadav, 2004; Dwivedi et al., 2010
Heat	H77/833-2, H77/29-2, CVJ 2-5-3-1-3, 77/371XBSECT CP1, 96AC-93, 1305, 77/371, Togo II, 99HS-18, G73107, 77/371	Yadav et al., 2014
Salinity	ICMB 02111, ICMB 94555, ICMB 95333, ICMB 00888, ICMB 01222, ICMP 451, IP 3732, IP 3757, IP8210, and PRLT 2/89-33, 10876 and 10878 (Sudan), 18406 and 18570 (Namibia), and ICMV93753 and ICMV 94474 (India); 863-B, CZI 98-11, CZI 9621, HTP 94/54	Ali et al., 2004; Dwivedi et al., 2010
**Biotic stress**	IP #16438 and 16762; P 310-17 and P 1449-3; IP18292; IP18293;700651; ICML #12, 13, 14, 15, 16, and 22; ICMP #312, 423, and 85410; 7042S; 841A; IP #9, 55, 104, 253, 262, 336, 346, 498, 545, and 558; landrace such as Ardi-Beniya Ka Bas, Dhodsar local and Desi Bajri-Chomu	Singh et al., 1997; Khairwal and Yadav, 2005; Thakur et al., 2006; Sharma et al., 2007
Downy mildew (*Sclerospora graminicola*)	ICMPE #13-6-30, 134-6-9, 134-6-34, 13-6-27, 37, and 71;ICML #1, 2, 3, 4, 5, 6, 7, 8, 9, 10; ICMA #91333, 91444, and 91555; ICMA 92666 and ICMB 92666 (resistant to ergot, smut, and downy mildew)	Thakur et al., 1982; Willingale et al., 1986; Thakur and King, 1988a,b; Thakur et al., 1992; Rai et al., 1998b; Khairwal and Yadav, 2005
Ergot (*Claviceps fusiformis*)	ExB 46-1-2-S-2, ExB 112-1-S-1-1, ICMV 8282, ICMV8283; ICMA 88006A and ICMA 88006B (resistant to smut and downy mildew);ICMA#91333, 91444 and 91555; 44 accessions selected from the screening of 1747 germplasm; ICML #5–10; ICMPS #100-5-1, 700-1-5-4, 900-1-4-1, 900-3-1, 900-9-3, 1300-2-1-2, 1400-1-6-2, 1600-2-4, 1500-7-3-2, 1800-3-1-2, and 2000-5-2; SSC FS 252-S-4, ICI 7517-S-1, ExB 132-2-S-5-2-DM-1, and P-489-S-3; SSC 46-2-2-1, SC 77-7-2-3-1, SSC 18-7-3-1	Thakur et al., 1983; Thakur and King, 1988c; Yadav and Duhan, 1996; Rai et al., 1998a; Khairwal and Yadav, 2005
Smut (*Moesziomyces penicillariae*)	ICML #5, 6, 7, 8, 9, and 10; ICML #17, 18, 19, 20 and 21; Tif leaf 3; Tift 3 (PI 547035) and Tift 4 (PI 547036); Tift 65 (resistant to rust and leaf spot)	Bourland, 1987; Thakur and King, 1988a; Wilson and Burton, 1991; Burton and Wilson, 1995; Hanna et al., 1997
Rust (*Puccinia* sp.)	IP #16438 and 16762; P 310-17 and P 1449-3; IP18292; IP18293;700651; ICML #12, 13, 14, 15, 16, and 22; ICMP #312, 423, and 85410; 7042S; 841A; IP #9, 55, 104, 253, 262, 336, 346, 498, 545, and 558; landrace such as Ardi-Beniya Ka Bas, Dhodsar local and Desi Bajri-Chomu	Singh et al., 1997; Khairwal and Yadav, 2005; Thakur et al., 2006; Sharma et al., 2007


#### Salinity

Salinity is also a major abiotic stress constraint for crops of arid and semi-arid regions of Africa and Asia where high surface evaporation, low precipitation, and poor irrigation habits result in the enhancement in the levels of soluble salts that make the ground water unavailable to plants (Minitzer, 1993; Goyal, 2004). Salinization becomes more severe in areas affected by intense drought and high-temperature stress resulting in increased upward movement of capillary water and water soluble salts to the root zone of plants (Szabolcs, 1990; Varallyay, 1994).

Though pearl millet is a crop with inbuilt capacity to withstand soil salinity and therefore is capable of cultivation in saline lands for grain and forage production, salinity also acts as a significant abiotic constraint for its cultivation in several areas of Africa and India, with more intense effects in the West Asia and North Asia (WANA) zones of Central Asia. As compared to other cereal crops only limited information has been available on response to soil salinity in pearl millet. Reduced shoot N content and increased K^+^ and Na^+^ content is usually associated with salinity tolerance in pearl millet (Dwivedi et al., 2012). According to Krishnamurthy et al. (2007), shoot biomass ratio associated with salt tolerance and shoot Na^+^ concentration could be used as potential selection criteria for screening of pearl millet germplasm at vegetative stage. ICRISAT has carried out some basic research on salinity tolerance of pearl millet in collaboration with the International Centre for Biosaline Agriculture (ICAB) along with NARS partners in both India and WANA region (Dwivedi et al., 2012). For the screening of salinity tolerant breeding materials of pearl millet, pot culture method has been followed in the salinity affected fields. Screening of pearl millet germplasm has resulted in the development of advanced breeding materials, improved population including OPVs, gene pools and composites, parental lines of potential hybrids, and germplasm accessions with high grain and forage yield presumably with a high degree of salinity tolerance (**Table 3**). In the short-to-medium terms, some of these materials can be released for cultivation after extensive validation of their yield performances at on-farm trials. Working on these lines, a pearl millet variety “HASHAKI I” has been identified for release in Uzbekistan in 2012 as a high-forage variety for salt-affected areas. The identified salinity-tolerant pearl millet lines should be utilized in breeding programs to develop salinity-tolerant locally adapted cultivars (both OPVs and hybrids). This will enable farmers in salt-affected areas to adopt and grow a new crop such as pearl millet in lands that otherwise are fallow most of the years (Yadav et al., 2012).

#### High Temperature

Most of the growth processes of pearl millet like rate of seed germination, coleoptile elongation, rate of photosynthesis, etc. requires optimum temperature of about 35°C indicating that it is adapted to the hot arid zones of Sahel and many parts of India (Gracia-Huidobro et al., 1985) where mid-day surface temperature could exceed 45°C (Singh et al., 1993). While temperature beyond 35°C could be detrimental to the development of most of the other cereal crops, pearl millet could thrive in hot environmental conditions and maintain their optimal growth and yield potential (Yadav et al., 2010).

In order to understand the effects of temperature on seed germination, supra-optimal effects of temperature have been studied (Yadav et al., 2010). It was found that seed germination in pearl millet usually occurs at 35°C till 45°C but decreases at 47°C and almost stop at 50°C (Garcia-Huidobro et al., 1982). Further, the high temperature of seedbed has been established as a major cause of poor plant stand in pearl millet (Ong, 1983a,b). It was also indicated from several field studies in Sahel region that pearl millet seedlings are most vulnerable to high temperatures during the first 10 days of sowing (Stomph, 1990). Since it is difficult to control high soil surface temperature by cultural methods, it becomes essential to identify genetic variations in pearl millet germplasm for tolerance to high-temperature seedbeds. H 77/833-2, an elite pearl millet inbred line, hybrids of which are widely used by farmers in north-western India, is sensitive to terminal drought stress but tolerant to high-temperature stress (Yadav et al., 2014). This cultivar has been used as one of the parents (recurrent) to generate a set of mapping population (NILs) to map QTLs for terminal DT of grain and stover yield as well as their component traits in pearl millet (Yadav R.S. et al., 2003, 2004; Hash et al., 2006b).

### Resistance to Biotic Stresses

Like other cereal crops, pearl millet is also prone to various biotic stresses including fungal, viral, and bacterial infections. Fungal infections including downy mildew, rust, smut, blast, and ergot are considered to impact pearl millet production and yield more severely than any other plant disease causing pathogens. Downy mildew or ‘green ear’ caused by *Sclerospora graminicola* (Sacc. Schroet.) is one of the most devastating diseases of pearl millet causing maximum yield loss in India and Africa (Kumar et al., 2012). Downy mildew attacks panicles and their effects may range from mild symptoms to catastrophes when large fields have been destroyed. The disease was first reported in India and was considered of minor importance till 1970 when HB3, a popular Indian pearl millet hybrid (Singh, 1995) suffered severe yield loss from approximately 8.2 million metric tons in 1970–1971 to 4.6 million metric tons in 1971–1972 due to downy mildew epidemic (Dwivedi et al., 2012; Kumar et al., 2012). Blast (*Pyricularia grisea*) and rust (*Puccinia substriata* var. *indica*) are other common fungal diseases affecting pearl millet fodder and grain production. Blast is a foliar disease while ergot (*Claviceps* spp.) and smut (*Moesziomyces penicillariae*) are tissue-specific diseases and mainly infect ovary part of the plant (Thakur and Williams, 1980). A combination of various disease management practices can be helpful in combating fungal disease including cultural methods, chemical methods, and host–plant resistance (Williams et al., 1981; Singh and Gopinath, 1985). Further identification and utilization of new genes for host plant resistance for developing varieties resistant to biotic stresses are very important. It can be achieved by developing precise phenotyping methods, exploiting natural genetic variations among germplasm, and with a detailed understanding of pathogen variability, as well as molecular and genetic mechanisms of resistance. At different centers of ICRISAT various techniques and resources have been refined for the identification and screening of virulent traits for downy mildew, rust, smut, and blast and ergot diseases. Furthermore effective phenotypic screens have been developed for downy mildew (Singh et al., 1997; Jones et al., 2002; Thakur et al., 2008), ergot (Thakur et al., 1982), rust (Singh et al., 1997), and smut (Thakur and King, 1988b) in pearl millet by ICRISAT and other research organizations. Pearl millet germplasm have also been extensively evaluated for resistance to major fungal diseases and several sources of resistance to downy mildew, blast, rust, smut and ergot have been identified. These sources of resistance have also been transferred into improved genetic backgrounds for developing resistant varieties (**Table 3**). Four widely cultivated OPVs from India namely, WC-C75, ICMS 7703, ICTP 8203, and ICMV 155 are resistant to downy mildew^8^ while ICMH 451 and Pusa 23 are popular downy mildew resistant hybrids (Singh, 1995). ICRISAT has also produced a top cross hybrid ICMH 88088 that possess a high level of downy mildew resistance and is also high yielding (Singh, 1995). A few downy mildew resistant varieties have also been developed by ICRISAT for Western Africa such as ICMV1 and ICMV2 for Senegal and IKMP2, IKMP3, and IKMV 8201 for Burkina Faso (Singh et al., 1993). Researchers have also gained some success toward identification of stable sources of disease resistance for smut (Thakur and King, 1988b), ergot (Thakur and King, 1988a), and rust (Singh et al., 1997) and some of these have also been used in breeding programs for enhanced disease resistance (Hash et al., 1999, 2006a).

However, in the last 10 years, the scenario has been changed for downy mildew as well as for other fungal diseases globally. Development and commercialization of new hybrid cultivars have also given rise to new strains of downy mildew pathogens (about 20 new virulent pathotypes of *S. graminicola* have been screened so far) (Thakur et al., 2011) and in coming years more new virulent strains could be identified. Further host-directed evolution of pathogenic variation has rendered downy mildew resistance rather elusive in Indian pearl millet inbred lines and hybrid cultivars (Thakur et al., 1992). Similarly, in the last few years, blast and rust disease outbreaks have been severe in some states of India. Therefore, it becomes essential to understand the pathogen variability, its mechanism of action and genetics of host plant resistance for the formulation of effective strategies for crop improvement against biotic stresses. Resistance to downy mildew shows dominance over susceptibility, additive or recessive traits, however, partial host plant resistance is controlled by one or more genes along with some modifiers (Hash and Witcombe, 2001; Breese et al., 2002; Dwivedi et al., 2012). Six major downy mildew pathotypes have been reported in India (Thakur et al., 2006). Sudisha et al. (2009) used Inter-simple sequence repeat (ISSR) primers to characterize pathogen variability; while 27 downy mildew isolates were grouped into six major pathotypes using 20 rapid amplified polymorphic DNA (RAPD) and 19 ISSR markers (Jogaiah et al., 2008). Similarly, 46 isolates of downy mildew could be classified into 21 pathotypes on the basis of disease incidence, latent period and virulence index, with pathotype P11 found to be most virulent (Sharma et al., 2010). In another study, 48 pearl millet inbred lines were evaluated against nine diverse *S. graminicola* isolates obtained from five different geographical locations in India which confirmed that gene pyramiding could enhance resistance to diverse isolates of downy mildew (Hash et al., 2006a). In a very recent study 39 native endophytic actinomycetes isolates from pearl millet roots were evaluated for their proteolytic action against downy mildew, of which seven strains could directly suppress sporangium formation in *S. graminicola* suggesting them as potential effective biocontrol agents (Jogaiah et al., 2016). Hence, despite the impressive progress toward managing various fungal diseases in pearl millet, there is still a lot of scope toward developing strategies for better management of diseases with the focus primarily on virulence monitoring, identification and characterization of newer isolates and developing resistant hybrids/cultivars for commercial cultivation.

## Genetic and Genomic Resources

### Markers, Genetic Linkage Maps, and Trait-Genetics for Marker-Assisted Breeding

DNA-based molecular markers, genetic linkage maps, and sequence information are essential genomic resources to carry out genetic studies or marker-aided breeding in any crop. Though the discovery of molecular markers and construction of genetic maps in millets lag far behind those of staple cereals, pearl millet reportedly has comparatively substantial stock of genetic and genomic resources in the form of DNA-based markers, mapping populations or linkage maps (Lata et al., 2013; Lata, 2015). In pearl millet various DNA-based molecular markers such as AFLP, RFLP, RAPD, expressed sequence tag-based (EST) markers, sequence-tagged sites (STSs), simple sequence repeat (SSRs/microsatellites), DArTs, CISP and SNP have been developed to distinguish genetic variability, linkage map analysis and marker assisted screening to expedite the breeding programs (Liu et al., 1994; Devos et al., 1995; Allouis et al., 2001; Qi et al., 2004; Supriya et al., 2011; Sehgal et al., 2012; Lata, 2015) (**Table 4**). Molecular markers help in evaluating genetic differences in germplasm collections for appropriate selection of mating parents for hybrid breeding, studying population structure, and analysis of QTLs for abiotic and biotic stress resistance. Pearl millet exhibits a very high level of DNA marker polymorphism even between elite inbred parental lines of popular hybrids (Vadez et al., 2012).

**Table 4:** Summery of DNA based markers available in pearl millet related abiotic and biotic stress tolerance.

DNA markers	Reference
Thirty seven SSRs and CSIP markers developed, representing all seven linkage groups analyzed under both well- watered and drought conditions,22 SNPs and 3 InDels for abiotic stresses	Sehgal et al., 2015
ISSR derived SCAR marker developed for downy mildew (DM) resistance in pearl millet and linked with DM resistance linkage group with genetic linkage distance of 0.72 cM	Jogaiah et al., 2014
Seventy five SNPs and CISP developed from available expressed sequence tags (ESTs) using parents of two mapping populations for 18 genes	Sehgal et al., 2012
More than 100 polymorphic EST-SSR markers developed and mapped in one or more of four pearl millet RIL populations	Rajaram et al., 2010, 2013
250–280 DArT markers were analyzed for polymorphic in each of three pearl millet RIL populations	Senthilvel et al., 2010
Eleven of 31 finger millet EST-derived SSR primer pairs detected polymorpism in pearl millet	Arya et al., 2009
Four EST-SSRs and nine CISPs detecting polymorphism in one or more of four pearl millet biparental mapping populations	Yadav et al., 2008
A set of 21 polymorphic EST-SSRs and six genomic SSRs	Senthilvel et al., 2008
Nineteen EST-derived SSR primer pairs, of which 11 gave amplification products and four were show polymorphism on agarose gels	Yadav et al., 2007
Sixteen EST-derived polymorphic SSRs	Mariac et al., 2006
SSCP-SNP primer pairs developed by comparison of rice and pearl millet EST sequences	Bertin et al., 2005
Thirty six SSRs derived from genomic library	Qi et al., 2004
Eighteen SSRs derived from genomic library	Allouis et al., 2001; Budak et al., 2003


RFLP-based molecular markers were developed in pearl millet by Liu et al. (1992, 1994) and were mapped over 180 loci ranged approximately 350 cM in seven linkage groups, one extra couplet, and other was a free-floating point and later was used to map QTLs for resistance to downy mildew in pearl millet (Jones et al., 1995). A set of 21 polymorphic EST-based SSRs markers and six genomic markers were developed in pearl millet and were tested to detect polymorphism across 11 pairs of pearl millet mapping population parental lines (Senthilvel et al., 2010). These EST-based markers are also used for marker assisted breeding and crop improvement programs in pearl millet at ICRISAT. Senthilvel et al. (2010) also reported the development of an array of about 6900 DArT clones using PstI/BanII complexity reduction, and identification of a total of 256–277 polymorphic DArT markers in three RILs of pearl millet. A total of 574 polymorphic DArT markers were identified in a set of 24 genetically diverse pearl millet inbreds from 7000 DArT clones obtained from 95 diverse genotypes using PstI/BanII complexity reduction (Supriya et al., 2011). With the help of DArT markers, comparative genomics and genome organization studies can be easily done and the cost of marker-assisted backcrossing (MABC) is also low as compared to others. Sehgal et al. (2012) used available pearl millet ESTs and developed 75 SNP and CISP markers and demonstrated their use in identifying candidate genes underlying a major DT-QTL using four genotypes namely, H 77/833-2, PRLT 2/89-33, ICMR 01029 and ICMR 01004, representing parents of two mapping populations. There is another recent research on identification of 83,875 SNP markers using genotyping by sequencing (GBS) of PstI-MspI reduced representation libraries in 500 genotypes of pearl millet, comprised of 252 global accessions and 248 Senegalese landraces, which exhibited high genetic diversity relative to other genotypes of Africa and Asia (Hu et al., 2015). Further ISSR-derived sequence characterized amplified region (SCAR) markers were developed to screen out polymorphism in a pair of pearl millet genotypes ICMR-01007 (P1) and ICMR-01004 (P2) and their population for downy mildew resistance. A polymorphic band of 1.4 kb size was produced in ISMR-01004 variety only from a single primer set ISSR-22 and further PCR amplification of these polymorphic band was found to be tightly linked to downy mildew resistant linkage group (χ^2^ 3:1 = 0.86, *P* < 0.01) with a genetic distance of 0.72 cM. This SCAR marker was further validated in different Asian and African pearl millet cultivars and the result showed that the marker was associated with downy mildew disease resistant genotypes only (Jogaiah et al., 2014). Further, Ambawat et al. (2016) constructed a linkage map comprising 286 loci (229 DArT and 57 SSR markers) through the genotyping of 256 DArT and 70 SSR markers on 168 F_7_ RILs derived from the cross between 81B-P6 and ICMP 451-P8. Using these markers, QTL for rust resistance have been mapped on LG1 with LOD score of 27.

These molecular markers have been used to construct a pearl millet genetic linkage map (Liu et al., 1994) and to identify and map QTLs for terminal drought (Yadav et al., 2002), reduced salt uptake (Sharma et al., 2010, 2014), grain and stover yield (Yadav et al., 2002; Yadav R.S. et al., 2003, 2004), and for downy mildew resistance (Jones et al., 1995, 2002; Breese et al., 2002; Gulia, 2004; Gulia et al., 2007), rust and blast resistance (Morgan et al., 1998). These genetic tools have also been used for diversity assessment (Liu et al., 1992; Bhattacharjee et al., 2002), studying recombination rates (Busso et al., 1995; Liu et al., 1996), analyzing domestication syndrome (Poncet et al., 2000, 2002) and comparative genetics (Devos et al., 1998; Devos and Gale, 2000). The present pearl millet genetic linkage map covers approximately 700 cM distributed across the seven linkage groups as expected, and at least one pair of a free-floating RFLP-linked marker, however, telomeric regions of chromosomes have yet not been mapped (Vadez et al., 2012). Intriguingly these DNA marker-based linkage groups could not be distinctly linked with the pearl millet chromosome map (Kaul and Sidhu, 1997) developed using morphological markers (Kumar and Andrews, 1993) and conventional cytogenetic methods (Jauhar and Hanna, 1998) suggesting the scope of more focused research in this direction. Further identification of minor QTLs using conventional mapping population is thought to be a complex process partly due to the interaction(s) between QTLs and/or due to the strong overshadowing effects of major QTLs. Kumari et al. (2014) developed a set of chromosome segment substitution lines (CSSLs) for the identification of minor QTLs by introgressing overlapping chromosome segments from 863B into ICMB 841 background for use in QTL detection, fine mapping, and complex trait mechanism studies. Advanced generation backcross progenies (1492) that were expected to provide coverage across each of the seven pearl millet linkage groups, were genotyped at 74 marker loci involving SSRS, SSCP-SNP, and STSs markers, identifying 124 segment introgression homozygotes. Recently GBS approach is gaining popularity for the development of genetic markers and high density linkage map for species lacking reference genome (Punnuri et al., 2016). The authors have developed 96-plex *Ape*KI GBS library from the 186 RILs and from their parents (‘Tift 99D2B1’ and ‘Tift 454’) and F1 population. DNA of these populations was sequenced and the results were used for the development of reference genetic map using 150 RILs that contained a total of 16,650 SNPs and 333,567 sequence tags spread across all seven pearl millet chromosomes. The final map has a genetic distance of 716.7 cM with 23.23/cM overall average density of SNPs and 1.66 unique linkage bins per cM. This map was further used in mapping QTLs for flowering and resistance to *Pyricularia* leaf spot disease caused by [*Pyricularia grisea* (Cke.) Sacc.] in pearl millet.

It has also been proposed that pearl millet genome has undergone several structural rearrangements (Devos and Gale, 2000) that could be possibly associated with its evolution and adaptation in severe abiotic stresses and nutrient deficient soils although until now marker relationships are almost collinear across all the 10 mapping populations developed and mapped (Liu et al., 1996; Azhaguvel, 2001; Kolesnikova, 2001; Vadez et al., 2012).

### Identification and Functional Validation of Genes Associated with Abiotic and Biotic Stress Tolerance

In last few years, the scientific community has gained some success toward understanding the physiological basis of stress tolerance and open new avenues for the development of more drought- tolerant pearl millet inbred lines and hybrids using MABC (Serraj et al., 2005). In pearl millet several QTLs have been identified and mapped to downy mildew, DT and yield components (**Table 5**), however linkage analysis in most of these studies suggest presence of genes/QTLs at a distance of 10–40 cM from the closest markers making it difficult for MAB or functional validation of candidate genes (Dwivedi et al., 2012). Until now only genes associated with a major DT-QTL have been identified in this crop (Sehgal et al., 2012). Moreover, it is apparent that in comparison to other cereal crops, little attention has been given toward identification and characterization of candidate genes for various abiotic and biotic stress resistances in pearl millet. There have been only a few reports on transcriptome analysis in pearl millet for delineating the mechanism of abiotic stress tolerance till date (Mishra et al., 2007; Choudhary et al., 2015). Both studies were done using suppression subtractive hybridization (SSH) technique. Mishra et al. (2007) reported the identification of about 2,494 differentially regulated transcripts in response to drought, salinity, and cold stress indicating the existence of a complex gene regulatory network in this stress tolerant crop. While Choudhary et al. (2015) reported transcript profiling of 22-days old seedlings of *P. glaucum* subjected to 30% PEG for different time periods ranging from 0.5 to 48 h that led to the identification of 745 ESTs in response to drought stress which could be helpful in unraveling the molecular basis underlying tolerance to drought stress. In another study, transcriptome analysis using 454 Roche NGS tool was done to understand the mechanisms underlying resistance to downy mildew in pearl millet (Kulkarni et al., 2016). A total of 1396 and 936 up- and down- regulated transcripts in resistant inoculated/resistant control, and 1000 and 1591 transcripts in susceptible inoculated/susceptible control were identified. The study revealed the up-regulation of phenylpropanoid pathway genes in resistant genotype and also evoked potentials of hypersensitive response and systemic acquired resistance as possible defense mechanisms operating against downy mildew infection in pearl millet. A comprehensive pearl millet transcriptome assembly (∼43 Mbp) has also been developed by integrating data from three independent investigations carried out by Zeng et al. (2011) and Rajaram et al. (2013) and is comprised of about 69, 398 tentative assembly contigs^9^. This transcriptome assembly also includes pearl millet ovule transcriptome data. Recently three studies have been published regarding identification and selection of suitable reference genes for assessment of gene expression through quantitative real-time (qRT)-PCR analysis under individual as well as multiple stress conditions (Saha and Blumwald, 2014; Reddy et al., 2015; Shivhare and Lata, 2016).

**Table 5:** Functional validation of important genes linked with biotic and abiotic stress response in pearl millet.

Gene	Gene function	Source	Plant/organism tested	Type of tolerance	Reference
VDAC	Voltage dependent anion channel	*Pennisetum glaucum*	yeast	Salt stress	Desai et al., 2006
Afp	Formation of antifungal protein AFP	*Aspergillus giganteus*	*Pennisetum glaucum*	Downy mildew resistance	Girgi et al., 2006
Rab 7	Small GTP- binding protien	*Pennisetum glaucum*	Tobacco	Abiotic stress	Agarwal et al., 2009
Hsc 70	Molecular chaperons	*Pennisetum glaucum*	*E. coli*	Abiotic stress	Reddy et al., 2010
HSP 90	Molecular chaperons	*Pennisetum glaucum*	*E. coli*	Abiotic stress	Reddy et al., 2010
LEA	Formation of late embryogenesis abundant protien	*Pennisetum glaucum*	*E. coli*	Heat and salinity stress	Reddy et al., 2012
NPR1	Non-expresser pathogenesis related gene	*Brassica juncea*	*Pennisetum glaucum*	Downy mildew	Ramineni et al., 2014
Class I Small Hsp	Molecular chaperons	*Pennisetum glaucum*	*E. coli*	Heat tolerance	Reddy et al., 2015
CC-NBS-LRR	Plant disease resistance gene	*Pennisetum glaucum*	*Pennisetum glaucum*	Downy mildew resistance	Veena et al., 2016


Improved transformation of millets linked to significant gene targets which may offer resistance to various biotic and abiotic stresses will play important role in crop improvement. Of late development of cultivars using genetic engineering approaches is gaining significance in plant biology and stress physiology. The understanding of mechanisms that regulate gene expression and the capacity to transfer important genes from other organisms into plants will expand the ways in which plants can be utilized. The use of newer approaches combining physiological, biochemical, molecular and genetic techniques should provide excellent results in near future. The task of generating transgenic cultivars is not only limited to the success of the transformation process but also the proper emphasis on incorporation of stress tolerance. Functional analysis *in vivo* is fundamental to transgenic technology which helps to investigate molecular mechanisms of biotic and abiotic stress regulation. Important methods to functionally validate candidate gene(s) for stress tolerance include overexpression, gene silencing or genome editing in a plant system. In pearl millet, the biolistic method for gene delivery has been frequently used for transformation (Ceasar and Ignacimuthu, 2009; Lata, 2015). There have been only a few reports on achieving stress tolerance through transgenic approaches in pearl millet (**Table 6**) as for example, Girgi et al. (2006), introduced an afp gene from the mold *Aspergillus giganteus*, encoding an antifungal protein AFP into pearl millet through particle bombardment. The transgenic plants showed a significant reduction in disease symptoms against rust and downy mildew infection as compared to control plants with an increase in disease resistance by up to 90%. Similarly, a *Brassica juncea* non-expressor of pathogenesis-related gene 1 (*BjNPR1*) has been stably integrated and expressed into a pearl millet male fertility restorer line ICMP451 through *Agrobacterium*-mediated transformation (Ramineni et al., 2014). However, until now there has been no report on the generation of abiotic stress tolerant transgenic pearl millet line. Therefore, it can be inferred that development of genetically engineered pearl millet varieties is still in nascent stage despite its economic importance and hence calls for a concerted effort for generation and evaluation of transgenic lines under various stress conditions.

**Table 6:** Quantitative trait loci (QTLs) associated with important traits under abiotic and biotic stresses in pearl millet.

QTLs	Linkage group	Associated trait	Reference
DT- QTL	LG-2	Terminal drought stress	Yadav et al., 2002; Yadav R.S. et al., 2004; Bidinger et al., 2005, 2007; Sehgal et al., 2009
DT- QTL	LG-2	Reduced salt uptake and	Sharma et al., 2011, 2014
GRYLD- QTL	LG-2	Drought tolerance in grain yield in early stress environments	Bidinger et al., 2007
GRYLD- QTL	LG-5	Drought tolerance in grain yield in early stress environments	Yadav R.S. et al., 2004
GRYLD- QTL	LG-3, LG-4, and LG-6	Drought tolerance in grain yield in late stress environments	Bidinger et al., 2007
DM-QTL	LG-1, LG-4	Downy mildew	Jones et al., 1995


### Pearl Millet Genome Sequencing

Though, in past few years crucial efforts have been made toward generating genomic resources in the form of molecular markers and genetic linkage maps in pearl millet but the collection is still not up to the mark as compared to other cereal crops. Hence, with the aim to generate sufficient genomic resources for augmenting breeding programs in pearl millet by sequencing pearl millet genome, a consortium of various international organizations namely ICRISAT; L’Institut de Recherché pour le Développement (IRD), France; the Indian Council of Agricultural Research (ICAR), India; the University of Georgia, USA; Cornell University, USA; University of Florida USA; L’Institut Sénégalais de Recherches Agricoles (ISRA); and Pioneer Overseas Corporation, India has been formed^10^. A global pearl millet reference genotype, Tift 23DB2B1 has been chosen to develop its draft genome sequence through whole genome shotgun and Bacterial artificial chromosome (BAC) sequence approaches. There have also been efforts to resequence around 993 pearl millet germplasm lines for validation. Hence, the rigorous sequencing efforts will be definitely helpful to the researchers working in crop genetics and breeding particularly pearl millet breeding.

## Conclusion and Future Perspectives

**FIGURE 1:**
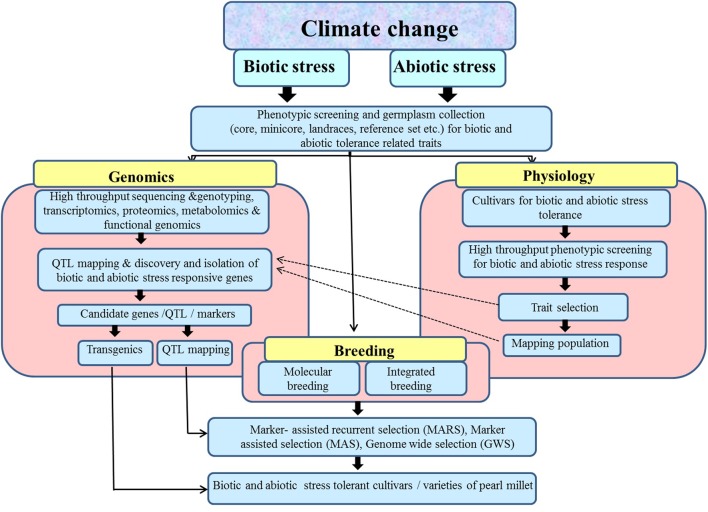
**A combined approach for integrating genomics, physiology, and breeding techniques for developing improved cultivars with enhanced abiotic and biotic stress resistance in pearl millet**.

In summary, pearl millet could serve as an excellent model for abiotic and biotic stress research owing to its hardy nature. Therefore, efforts should be focused on collection and characterization of pearl millet germplasm so that potential sources of genetic variation could be identified and incorporated in crop improvement programs. A concerted effort is also needed to develop and upgrade phenotypic screens for abiotic stress tolerance particularly high temperature, and biotic stress resistance such as downy mildew. There is a necessity for continuous monitoring of pathogen variability in pearl millet against various fungal pathogens. There is also a dire need to expedite the genetic and genomic resources of pearl millet for the development of more efficient genomic and sequenced based molecular markers for carrying out trait genetics and association mapping. This will help in identification and validation of candidate genes associated with important agronomic traits including abiotic stress tolerance and biotic stress resistance. The potential candidate genes identified through various transcriptomic and proteomic studies could be utilized for the development of cultivars with improved adaptability to survive harsh environmental conditions either through transgenic technology or MAB (**Figure 1**). It is hoped that with the completion and release of pearl millet genome sequence, the genetic and molecular studies in this otherwise naturally stress tolerant crop will progress swiftly. The advanced high throughput assays and next-generation sequencing could be used to resequence a large number of pearl millet germplasm. It will not only help in identification and tracking of natural genetic variations present within the germplasm collection but will also help in the discovery of genes and molecular markers associated with important agronomic traits, leading to newer opportunities for crop improvement in pearl millet and related crops.

## Author Contributions

CL and RS wrote and reviewed the manuscript. Both authors contributed equally.

## Conflict of Interest Statement

The authors declare that the research was conducted in the absence of any commercial or financial relationships that could be construed as a potential conflict of interest.

## Footnotes

1 http://exploreit.icrisat.org/page/pearl_millet/680/273

2 http://exploreit.icrisat.org/page/pearl_millet/680

3 http://www.aicpmip.res.in/pmnews2016.pdf

4 http://www.feedipedia.org/node/399

5 http://edis.ifas.ufl.edu/ag347

6 http://agritech.tnau.ac.in/seed_certification/seed_cm_pearl\%20miilets.html

7 http://www.aicpmip.res.in/pmbiology.pdf

8 http://agropedia.iitk.ac.in/content/downy-mildew-pearl-millet

9 http://www.ceg.icrisat.org/transcriptome.html

10 http://www.ceg.icrisat.org/ipgsc.html
